# Elevated Perinatal Depression during the COVID-19 Pandemic: A National Study among Jewish and Arab Women in Israel

**DOI:** 10.3390/jcm11020349

**Published:** 2022-01-11

**Authors:** Samira Alfayumi-Zeadna, Rena Bina, Drorit Levy, Rachel Merzbach, Atif Zeadna

**Affiliations:** 1 MAP Centre for Urban Health Solutions, Li Ka Shing Knowledge Institute, Michael’s Hospital, Unity Health Toronto, Toronto, ON M5B 1W8, Canada; 2 The Louis and Gabi Weisfeld School of Social Work, Bar Ilan University, Ramat Gan 5290002, Israel; Rena.Bina@biu.ac.il (R.B.); drorit.levy@biu.ac.il (D.L.); rachel.merzbach@live.biu.ac.il (R.M.); 3 Fertility and IVF Unit, Department of Obstetrics and Gynecology, Soroka University Medical Center, Faculty of Health Sciences, Ben-Gurion University of the Negev, Beer-Sheva 8410402, Israel; atifzeadna@gmail.com

**Keywords:** COVID-19, pandemic, perinatal, pregnancy, postpartum, depression

## Abstract

This study assessed prevalence of perinatal depression symptoms (PNDS) during the COVID-19 pandemic among Arab and Jewish women in Israel and identified COVID-19-related risk factors for PNDS, while comparing Arab and Jewish women. Sample included 730 perinatal women (604 Jewish and 126 Arab) aged 19–45 years, who filled out an online self-report questionnaire. The questionnaire assessed several areas: perinatal experiences and exposure to COVID-19, social support, and financial and emotional impact. PNDS was measured by the Edinburgh Postnatal Depression Scale (EPDS). Prevalence of PNDS (EPDS ≥ 10) in the entire study population was 40.0%. Prevalence among Arab women was significantly higher compared to Jewish women (58% vs. 36%, PV < 0.001). Higher PNDS were significantly associated with anxiety symptoms (GAD ≥ 10) (PV < 0.001), stress related to COVID-19 (PV < 0.001), adverse change in delivery of healthcare services (PV = 0.025), and unemployment (PV = 0.002). PNDS has elevated more than twofold during COVID-19 in Israel. Such high rates of PNDS may potentially negatively impact women, and fetal and child health development. This situation requires special attention from public health services and policy makers to provide support and mitigation strategies for pregnant and postpartum women in times of health crises.

## 1. Introduction

The COVID-19 pandemic has had many adverse impacts on the health and wellbeing of populations worldwide. Yet one of the most vulnerable groups deserving of close examination is that of perinatal women. The perinatal period is a time of heightened risk for women to develop emotional distress, e.g., depression. Not surprisingly, given that anxiety is a major risk factor for depression [1,2], several studies have shown that a high incidence of mental illness occurs during the perinatal period [3,4,5], putting perinatal women at even higher risk for onset or worsening of their mental health [6]. 

Indeed, global pre-pandemic rates of perinatal depression (PND) were reported to be 12–20% [1,2]. PND is associated with stressful life events or environments, existing or a history of mental health conditions, marital conflict, unplanned pregnancy, lack of social support, low socioeconomic status, and diagnosis of adverse pregnancy outcomes such as miscarriage, preterm delivery, low birth weight, and adverse maternal health conditions [7,8]. Untreated PND adversely impacts women’s health, infant outcomes, mother–infant bonding, and subsequent offspring physical and emotional health and cognitive development, and PND had, even before the pandemic, already emerged as a major public health concern [2,9,10,11]. 

However, the global COVID-19 pandemic has introduced unique challenges that subject pregnant and postpartum women to various physiological and psychosocial changes, making them even more prone to distress [12,13]. COVID-19 can attack lung cells, the immune system, and vascular, renal, and gastrointestinal cells, and may lead to severe respiratory distress syndrome [14]. Additionally, the extensive discussions in the media and social networks regarding pandemic-related health risk issues, including increased risks of miscarriage and fetal malformations [15], as well as potential mother–child transmission and increased risk of negative newborn outcomes [16,17], were found to contribute to elevated levels of anxiety and stress among perinatal women [18,19]. Other characteristics of the pandemic period, such as loneliness and isolation due to deprivation of social and family support, a reduction in maternity services, the limited availability of medical resources, restricted travel to hospital appointments, financial difficulties, domestic conflicts, and abuse, all of which have increased worldwide, were found to contribute to increasing the incidence of PND symptoms [12,20,21]. Rates of PND during COVID-19 were reported around 31–40% [12,22]. One study conducted in Canada during COVID-19, for example, showed increased PND and anxiety symptoms (37% and 57%, respectively) compared to similar pre-pandemic pregnancy cohorts [23]. 

### Perinatal Mental Health in Israel

Israel, with a multi-cultural population of approximately 9.291 million people, is comprised of a majority Jewish population and a large (21.1%) Arab minority [24]. Studies have shown that this minority population experiences socioeconomic and health inequalities at both the individual and neighborhood levels [25]. For instance, compared to the majority population, Arab society tends to have lower levels of education, income, and employment [26,27] as well as poorer self-rated health [28]. These inequalities have been attributed to insufficient infrastructure in Arab localities and longstanding discriminatory policies which themselves contribute to high stress [29], low social cohesion, high depression, anxiety, and intimate partner violence [25]. Indeed, findings of pre-pandemic studies examining perinatal period mental health have shown higher rates (16.3% to 43%) of depression among Arab women as compared to that (4.5–22.6%) in Jewish women [30,31,32,33,34,35].

Given the context of the COVID-19-pandemic-related psychological distress experienced by perinatal women and the short- and long-term effects suffered by them and their children, it is essential to explore the risk factors related to the COVID-19 pandemic that contribute to these adverse effects. Moreover, as Arab women in Israel are more vulnerable than their Jewish counterparts to psychological distress during the perinatal period and may be at even greater risk as a result of the spread of COVID-19 [36], in order to address the unique needs of each population it is important to explore the risk factors of mental health that are unique to COVID-19 in perinatal Arab and Jewish women. To the best of our knowledge, the current study is the first to assess the prevalence of perinatal depression symptoms (PNDS) during the COVID-19 pandemic in a nationwide sample of Israeli Arab and Jewish women with the aim of identifying pandemic-related PNDS risk factors.

## 2. Methods

### 2.1. Study Design and Participants

The population comprising this cross-sectional study consisted of women who were pregnant or up to six months postpartum. Study inclusion criteria included being 18 years of age or older, pregnant or a biological mother of a child six months old or younger, and Hebrew or Arabic speaking, as well as consenting to participate. The study was carried out from August 2020 to February 2021. Data from 972 participants were considered valid and, in this study, only women that completed the EPDS were included. Ultimately, data on 730 perinatal women (604 Jewish and 126 Arab) were included in the analysis.

### 2.2. Sample Size

No restrictions were set on participant enrollment. However, a representative sample size was calculated according to the previous year’s number of Israeli newborns. Thus, based on an α-level of 0.05, we estimated a minimum sample size of 300 participants.

### 2.3. Data Collection

Data for the current study were collected through an online, structured, self-report questionnaire in Hebrew and in Arabic. Participants were recruited through social media (i.e., Facebook, Instagram, and WhatsApp) as well as through the personal networks of colleagues and acquaintances of research team members. Participants were asked to click on the project’s website link and were then directed to the online questionnaire. Once there, they were asked to confirm a set of eligibility criteria regarding their age and pregnant or postpartum status. Next, they were asked for their consent to participate in the study after reading an electronic consent form presenting an overview of the study aims, content of the questions asked, potential risks and benefits, and ethical aspects. Finally, those who met the predefined inclusion criteria filled out the questionnaire, while those who did not were directed to a message thanking them for their interest and informing them of the required eligibility criteria for study participation. The questionnaire took approximately 20 min to complete.

### 2.4. Variables and Measures

#### 2.4.1. Outcome Measures

Perinatal depression symptoms (PNDS)

Perinatal depression symptoms (PNDS) were measured by the Edinburgh Postnatal Depression Scale (EPDS) [37], the most widely used self-report scale designed to identify women at risk for PNDS [38]. Indicating how women felt during the previous week, this 10-item scale assesses symptoms of sadness, anxiety, and thoughts about death. Scores range from 0 to 30, with the depression level being the sum score of all items and higher scores indicating greater symptom severity. As women who score ≥10 are considered at risk for PNDS [39,40], this cutoff score was chosen for use in the current study. Validated Hebrew and Arabic translated versions of the EPDS were also used. Furthermore, a reliability test revealed that Cronbach’s alpha was 0.87 for the entire study, 0.88 for Jewish women and 0.84 for Arab women.

#### 2.4.2. Independent Variable Measures 

Socio-Demographic characteristics

Demographic information included *ethnicity* (a dichotomous variable with two categories, Jewish and Arab, determined by mother’s self-identification), *age*, *marital status* (divorced, separated, widowed, or other), *educational level* (high school or below, above high school, or academic degree), *employment status* (Working full-time, part-time, or casually or Not working [unemployed, on maternity leave, retired, housewife, or other]), *woman’s income* (With income [working full time, working part-time, or paid maternity leave] or No income [unpaid maternity leave, unpaid temporary leave, looking for job, student, unemployed, stay-at-home caregiver]), *number of children* (one child, two children, and ≥3 children), *pregnancy status* (pregnant or postpartum), *gestational age,* and *baby’s age*.

*Anxiety symptoms* were evaluated using validated Hebrew and Arabic translated versions of the Generalized Anxiety Disorder Screener (GAD-7) [41], which is based on criteria from the Diagnostic and Statistical Manual of Mental Disorders (DSM)-IV and DSM-IV-TR [42]. The seven items on this questionnaire assess worry, tension, restlessness, and irritability. The GAD-7 score is calculated by assigning scores of 0, 1, 2, and 3 to the response categories of ‘not at all’, ‘several days’, ‘more than half the days’, and ‘nearly every day’, respectively, then adding together the scores for the seven questions. The GAD-7 total score ranges from 0 to 21, with anxiety level represented by the sum score of all items, and with higher scores reflecting proportionally greater anxiety. A score of ≥10 is considered a risk for anxiety symptoms [41,43] and was used as a cutoff score in the current study. A reliability test revealed that Cronbach’s alpha for this study was 0.85 for the entire study, 0.84 for Jewish women, and 0.83 for Arab women.

*Social support* was determined by the direct question, “Currently, how supported do you feel by your social network?” Answers given were based on a seven-point Likert scale ranging from “not supported” to “very supported.” The scale was dichotomized by the median score into high (≥median score) and low (<median score) social support. 

*Diagnosis of COVID-19*: two categories were included, positive to COVID-19 test and negative to COVID-19 test at any time during the pandemic.

*Stress related to COVID-19* was determined by a single question concerning overall level of stress related to the pandemic. Answers given were based on a seven-point Likert scale ranging from 1 (No stress) to 7 (Highly stressed). This is one of the most accurate of the Likert scales, respondent is given seven different options to best reflect their feeling. This scale was dichotomized by the median score into high (≥median score) and low (<median score).

*Changes in healthcare service delivery:* this was determined by a single question regarding any change in perinatal health service due to the COVID-19 outbreak. Answers given were based on a five-point Likert scale ranging from 1 (significantly improved) to 5 (significantly worsened). The options include two extremes, two intermediate, and one neutral opinion measuring agreement with and likelihood of changes in healthcare delivery. The scale was dichotomized into three categories (improved, no change, and adverse).

### 2.5. Statistical Analysis 

Statistical analyses were performed using SPSS software version 22. Bivariate analysis was performed to examine associations between the independent variables and PNDS using the Chi-square test and t-test. Multivariate logistic regression analyses were performed to identify risk factors for PNDS. All independent variables associated (*p <* 0.05) with PNDS in the bivariate analyses were included in the multivariate analysis. The OR and 95% confidence interval (95% CI) were computed. 

### 2.6. Ethical Considerations 

Ethical approval was obtained from Bar Ilan University’s ethics committee before the study commenced (062001/2). Electronic informed consent was obtained from all participants, and confidentiality related to all information provided has been ensured. Since the survey was distributed online, a debriefing procedure was made available; at the end of the survey, a list of up-to-date services and resources for emotional help was provided. 

## 3. Results

Table 1 presents the distribution of variables for the entire study population as well as the differences between the two ethnic groups. The total sample included 730 pregnant and postpartum women (82.7% Jewish and 17.3% Arab). Several significant differences were found to exist between the two ethnic groups. For example, compared to their Jewish counterparts, Arab women were a bit younger and had more children, a greater proportion of them were married, and they were more likely to report low social support. Jewish women had a higher level of education, and a greater proportion of them were employed and earning an income during the pandemic. Regarding medical factors, as can also be seen in Table 1, a higher proportion of Arab women had tested positive for COVID-19, while more Jewish women reported adverse changes in healthcare service delivery.

**Figure 1:**
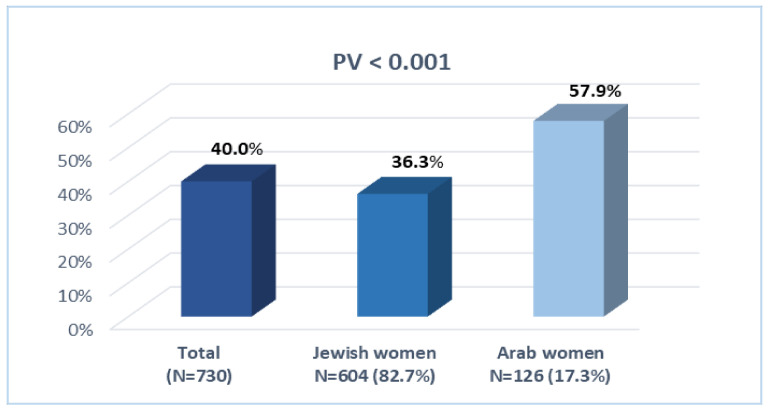
Prevalence of perinatal depression symptoms in total study sample and in Jewish and Arab women. *p* = significance level for the association between Arab and Jewish women (Chi-square test).

Figure 1 shows that the prevalence of PNDS in the total study population was 40.0%, with significant differences between Arab and Jewish women. While prevalence of PNDS among Jewish women was found to be 36.3%, among Arab women it was found to be 57.9%.

Bivariate analysis of PNDS risk factors, using EPDS ≥ 10 as a cutoff score for indicating presence of PNDS, showed a higher prevalence of PNDS among women who reported symptoms of anxiety (GAD ≥ 10), were unemployed, reported stress related to the COVID-19 pandemic, and reported adverse changes in healthcare service delivery (Table 2).

Table 3 presents the results of a multivariate logistic regression model comparing women with EPDS scores ≥ 10 to women who scored lower than 10. All variables significantly associated with PNDS in the bivariate analysis as well as the variables indicating significant differences between the two ethnic groups in Table 1 were included as independent variables in the multivariate analysis. Subsequent analysis revealed that the likelihood of Arab women experiencing PNDS was five times greater (OR = 5.0, 95%, confidence intervals (CI) = 2.7–9.2) than the odds of Jewish women experiencing PNDS. Furthermore, the likelihood of experiencing PNDS was found to be more than 21 times greater for women who reported anxiety symptoms (GAD ≥ 10) (OR = 21.8, 95%, CI = 11.2–58.7), four times greater for those who reported stress related to the COVID-19 pandemic (OR = 4.3, 95% CI = 3.0–6.4), and three times greater for those who were unemployed (OR = 2.7, 95% CI = 1.5–4.9), compared to those who did not report anxiety symptoms or stress and were employed, respectively. In addition, participants who reported adverse changes in healthcare service delivery were at greater risk for PNDS compared to participants who reported no change or positive change in the delivery of healthcare services. 

## 4. Discussion

The COVID-19 pandemic has dramatically affected the wellbeing and mental health of billions of people worldwide [44,45]. Changes in daily routine and restrictions in movement, together with the resulting confinement-related stress, financial insecurity, and burden of health concerns, have taken a heavy negative toll [46,47]. One of the most vulnerable groups impacted by these adverse circumstances is perinatal women at risk of PNDS [12,22]. The current study assessed the prevalence of PNDS in the Israeli Arab and Jewish population and the impact of several of its risk factors related to the COVID-19 pandemic. These two ethnic groups were found to differ significantly in terms of age, marital status, level of education, rate of employment, number of children, pregnancy status, social support, diagnosis of COVID-19, change in the delivery of healthcare services, and prevalence and level of PNDS (36.3% in Jewish and 57.9% in Arab women). 

It is interesting to note that the prevalence of PNDS found in Jewish women in Israel is similar to that found in women in other countries during the COVID-19 pandemic. For example, the prevalence in perinatal women was found to be 36.4% in the U.S [48], 39.2% in perinatal women in Qatar [49], 37% in pregnant women in Canada [23], and 34.0% in postpartum women in Turkey [50]. These findings indicate that PNDS incidence has more than doubled during the COVID-19 pandemic compared to its pre-pandemic rates [1,2]. 

Yet the current study found the PNDS rate in Israeli Arab women to be considerably higher than the 20.8% to 43% reported by studies conducted during pre-pandemic times [30,31,32,34,35]. This significant increase may well be associated with their societal circumstances. Arab women in Israel, comprising a minority population, have relatively low socioeconomic status [51] and less access to healthcare services and mental health services, and thus an especially low awareness of PND [52]. Likewise, a high prevalence of PNDS during the COVID-19 pandemic in other populations of minority women has been reported by previous studies. Qualitative research conducted in Northern California in minority pregnant and postpartum women found that those who reported PNDS faced individual, social, and healthcare service barriers to PND treatment [53]. Another study conducted in minority perinatal women with low-income status in the US showed that during the pandemic these women experienced increased stress and decreased social support [54]. 

Regarding the positive association between anxiety symptoms (GAD ≥ 10) and PNDS, the current study revealed that differences between Jewish and Arab women in scoring 10 or above on the GAD were not significant (13.7% and 12.2%, respectively). However, women who scored 10 or above on the GAD were more than 20 times more likely to report PNDS than those who scored GAD < 10. This positive association between PNDS and anxiety symptoms has been frequently reported [3,55,56,57]; in fact, having a history of anxiety disorders is one of the strongest predictors of PNDS [20,48]. 

Looking at other risk factors associated with pandemic-time PNDS addressed in the current study, three risk factors, i.e., unemployment, COVID-19-related stress, and negative changes in the delivery of healthcare services, in particular, were found to be dramatically reflective of these adverse times. It was found that 75% of Jewish women were employed compared to only 47.8% of Arab women. This rate of employment, while similar to the pre-pandemic rate for Arab women, is only a bit lower than the pre-pandemic rate (80%) for Jewish women [26]. The current study also showed that the likelihood of PNDS experienced by unemployed women was 2.7 times higher than that for employed women. Not surprisingly, unemployment is associated with food insecurity, domestic violence, and depression in pregnant and postpartum women [58,59]. Research has further suggested that poverty is a powerful predictor of depression and, as such, disproportionately affects low-income women [60]. 

Moreover, the current study found that stress related to COVID-19 affected both Jewish and Arab women, 38.7% and 32.2%, respectively. However, participants who were extremely concerned about stress related to COVID-19 constituted 61.8% of the group reporting PNDS. This group’s likelihood of PNDS was 4.6 times higher than that of study participants who reported relatively little concern about stress related to COVID-19 issues. Another study conducted in Israel during the pandemic revealed that Arab women were more anxious than Jewish women about several pandemic-related issues [36] such as being in public places, using public transportation, the need to attend pregnancy follow-up at hospitals or women’s health clinics, and delivery during the pandemic [36]. Some of the concerns raised by this study include how people of low socioeconomic status are characterized as being dependent on public transportation, avoiding public places, and having a lack of access to public health services. These findings correspond to the current study’s results, which indicate a higher PNDS prevalence among Arab women, an ethno-national minority in Israel characterized as having a low socioeconomic status [27].

Finally, the present study showed that a change in delivery of healthcare services in Israel during the pandemic was significantly associated with PNDS, i.e., participants reporting an adverse change were at significantly higher risk for PNDS (OR = 3.6). Substantial changes in the delivery of healthcare services have been made in several other countries as well, in an effort to minimize transmission risks [61]. Such changes include minimizing face-to-face care, physical-distancing restrictions, reducing care, limiting women’s escorts during appointments and childbirth, and restricting visitors to maternity wards [62,63]. Additional studies have reported that perinatal women experience high levels of concern about birth expectations, as well as report feelings of fear and loneliness [64], while postpartum women are concerned about breastfeeding and infant weight gain due to lack of face-to-face contact with health professionals [65].

The current study found no association between PNDS and education, women’s income status, social support, or diagnosis of COVID-19. By comparison, previous studies have shown that low education, lack of social support, and lower income are indeed risk factors for PNDS [30,66]. A partial explanation for this discrepancy may lay in the extreme stress that perinatal women have endured due to the pandemic. For example, during two periods of lockdown in Israel between March 2020 and February 2021, fear and anxiety were common, particularly to people at high risk for COVID-19 infection, such as pregnant women [67,68]. While in ordinary non-pandemic times education and social support may function as protective factors by enhancing feelings of self-efficacy [13,69], in times of pandemic outbreak, worldwide anxiety level, particularly that of perinatal women, may be elevated to such an unprecedented level that education may no longer serve as a protective factor. Furthermore, as a result of lockdowns and social distancing, the pre-pandemic face-to-face support women were accustomed to receiving may not be available [70]. Therefore, they may be disappointed with the change in the way social support has in recent pandemic times been delivered. Further studies are necessary to examine and further understand these issues; regardless, these findings suggest there exists a considerable challenge to healthcare services to implement screening techniques that identify and implement programs to support women who suffer from PND.

### Limitations

The current study has some limitations. First, because of its cross-sectional design, no causal effects can be implied between its independent variables and PNDS. In addition, data were collected through an online survey and based on participants’ self-reports, a procedure which might result in self-report bias. Another limitation is the lack of screening for psychiatric conditions. Since participants are not physically in a face-to-face setting, it was impossible to conduct a clinical diagnosis of depression by a physician. However, at the end of the questionnaire, we provided information about seeking professional consultation for PND. In addition, the questionnaire for assessing anxiety disorders (GAD) refers only to general anxiety and in future studies, it might be advisable to include instruments that assess other types of anxiety disorders such as panic disorder and social phobia. Furthermore, the group of study participants did not include women under the age of 18. Thus, the current study may not represent a comprehensive range of mental health needs of perinatal women, especially those who may be disproportionately affected, e.g., with limited internet access or pregnant teens, by the pandemic. Nonetheless, it provides important insights into the state of PND and its associated risk factors in Israeli Arab and Jewish women during the COVID-19 pandemic.

## 5. Conclusions

To the best of our knowledge, this is the first study conducted on a nationwide sample of Israeli Jewish and Arab women assessing the prevalence of PNDS and COVID-19-related PNDS risk factors. The study highlights the phenomenon of elevated PND symptoms in pregnant and postpartum women, one of the most vulnerable groups in the population, during the pandemic. Study findings also point to a high prevalence of PNDS in Arab and Jewish women, 57.9% and 36.3%, respectively. Such high rates of PNDS have potentially negative implications not only for women, but also for fetal health and development; this alarming situation requires special attention from public health services. Most of the risk factors, including elevated rates of anxiety (GAD > 10), pandemic-related stress, unemployment, and negative changes in healthcare service delivery, elucidated by the current study can be directly attributed to the pandemic. However, it must be noted that one prominent risk factor for PNDS is ethnic identity. Arab women in general have relatively poor healthcare service access and a low awareness of PND, and reside in low-income households badly affected by the pandemic. Given the well-established evidence of how PND affects the wellbeing of women and their families, as well as the development of their offspring, it has proven crucial, especially during the pandemic, to screen, prevent, monitor, and target for treatment a range of mental health symptoms and emotional wellbeing. It would be advisable to make available to women during their perinatal period a variety of intervention programs, e.g., online therapy, adapted to pandemic periods. We also suggest preventive interventions before or early during pregnancy that improve sense of social support, involved parenting skills, perinatal education with follow-up, and increase knowledge about PND and improve access to healthcare, might contribute to PND prevention especially in women at risk and the disadvantaged population. This includes preventive interventions that taking place in primary care, perinatal obstetrics/gynecology, pediatric clinics, or other community settings. Additionally, health policy makers and health organizations might notify pediatric providers that children of depressed mothers are at risk of developmental and behavioral problems and suggest conducting a regular developmental evaluation of the child, offer guidance, and refer them early for more comprehensive assessment and management of developmental and behavioral disorders.

## Figures and Tables

**Table 1:** Sociodemographic characteristics of Arab and Jewish women (Total N = 730).

	Total 730 (100%)	Jewish Women 604 (82.7%)	Arab Women 126 (17.3%)	
Variables	*N* (%)	*N* (%)	*N* (%)	PV
Age (Mean (SD) Range)	31.5 (4.9), 19–45	31.9 (5.0), 19–45	29.9 (3.9), 21–41	<0.000
Education				<0.000
Academic degree	595 (81.5)	524 (89.1)	71 (57.7)	
Non-Academic degree	116 (15.9)	64 (10.9)	52 (42.3)	
Marital status				0.005
Married	674 (94.8)	554 (93.7)	120 (100%)	
Unmarried	37 (5.2)	37 (6.3)	0	
Employment status				0.009
Employed	613 (85.7)	525 (85.6)	25 (22.1)	
Unemployed	102 (14.3)	77 (12.8)	88 (78.0)	
Woman’s income				<0.000
With income	490 (71.4)	427 (75.0)	43 (47.8)	
No income	196 (28.6)	149 (25.0)	47 (52.2)	
Number of children				<0.000
0	38 (5.7)	18 (3.3)	20 (17.2)	
1–2	418 (62.5)	366 (66.2)	52 (44.8)	
≥3	213 (31.8)	169 (30.6)	44 (37.9)	
Pregnancy status				0.001
Pregnant	309 (42.3)	239 (39.6)	70 (55.6)	
Postpartum	421 (57.7)	365 (60.4)	56 (44.4)	
Gestational age (Mean (SD) Range)	26.0 (9.1), 4–41	25.7 (9.2), 14–41	27.0 (7.9), 12–38	0.297
Baby’s age (weeks) (Mean (SD)Range)	13.2(7.2), 0–30	13.6 (7.1), 0–30	11.3 (6.1), 4–24	0.017
Anxiety symptoms				0.659
GAD < 10	606 (86.6)	498 (82.2)	108 (87.8)	
GAD ≥ 10	94 (13.4)	79 (13.7)	15 (12.2)	
Social support				0.011
Low	358 (49.7)	312 (51.7)	46 (39.0)	
High	363 (50.3)	291 (48.3)	72 (61.0)	
Diagnosis of COVID-19				<0.000
Positive	682 (94.1)	581 (96.4)	101 (83.5)	
Negative	42 (5.8)	22 (3.6)	20 (16.5)	
Stress related to COVID-19				0.157
Low	450 (62.1)	368 (60.9)	82 (67.8)	
High	236 (39.1)	251 (38.7)	39 (32.2)	
Changes in healthcare service delivery				<0.000
Positive change	27 (3.8)	12 (2.1)	15 (12.4)	
No change	398 (56.6)	321 (55.2)	77 (63.6)	
Adverse change	278 (39.5)	249 (42.8)	29 (24.0)	

**Table 2:** Individual characteristics and risk factors related to COVID-19 and associations with PNDS (N = 730).

	EPDS < 10 *N* = 438 (60.0%)	EPDS ≥ 10 *N* = 292 (40.0%)	
Variables	*N* (%)	*N* (%)	PV
Ethnicity			<0.001
Jewish	385 (63.7)	219 (36.3)	
Arab	53 (42.1)	73 (57.9)	
Age (Mean (SD) Range)	31.60 (4.82), 21–48	31.49 (4.98), 19–46	0.788
Education			0.408
Academic degree	363 (61.0)	232 (39.0)	
Non-Academic degree	66 (56.9)	50 (43.1)	
Marital status			0.575
Married	406 (60.2)	268 (39.8)	
Unmarried	24 (64.9)	13 (35.1)	
Employment status			0.001
Employed	386 (63)	227 (37)	
Unemployed	46 (45.1)	56 (54.9)	
Woman’s income			0.194
With income	267 (62.5)	160 (37.5)	
No income	149 (57.5)	110 (42.5)	
Number of children			0.512
0	23 (60.5)	15 (39.5)	
1–2	245 (58.6)	173 (41.4)	
≥3	135 (63.4)	78 (36.6)	
Pregnancy status			0.603
Pregnant	182 (58.9)	127 (41.1)	
Postpartum	256 (60.8)	165 (39.2)	
Gestational age (Mean (SD) Range)	26.48 (9.17), 6–40	25.17 (8.74), 6–40	0.222
Age of baby (weeks) (Mean (SD) Range)	13.10 (6.95), 0–29	13.75 (7.19), 1–29	0.353
Anxiety symptoms			
GAD < 10	413 (68.2)	193 (31.8)	<0.000
GAD ≥ 10	6 (6.4)	88 (93.6)	
Social support			0.649
Low	213 (59.5)	145 (40.5)	
High	222 (61.2)	141 (38.8)	
Diagnosis of COVID-19			0.661
Negative	413 (60.6)	269 (39.4)	
Positive	24 (57.1)	18 (42.9)	
Stress related to COVID-19			0.000
Low	333 (74)	117 (26)	
High	105 (38.2)	170 (61.8)	
Changes in healthcare service delivery			0.003
Positive change	14 (51.9)	13 (48.1)	
No change	262 (65.8)	136 (34.2)	
Adverse change	148 (53.2)	130 (46.8)	

**Table 3:** Factors associated with PNDS (EPDS ≥ 10) as per multivariate logistic regression analysis.

Variable	OR (95%CI)	PV
Ethnicity		<0.001
Jewish	1 (ref)	
Arab	5.0 (2.7–9.2)	
Anxiety symptoms		<0.001
GAD < 10	1	
GAD ≥ 10	21.8 (11.2–58.7)	
Employment status		0.002
Employed	1	
Unemployed	2.7 (1.5–4.9)	
Woman’s income		0.166
With income	1	
No income	1.4 (0.8–2.4)	
Education		0.092
Academic degree	1	
Non-Academic degree	1.7 (0.9–3.3)	
Stress related to COVID-19 pandemic		<0.001
Low	1	
High	4.3 (3.0–6.4)	
Changes in healthcare service delivery		
Positive change	1	
No change	2.2 (0.6–8.6)	0.050
Adverse change	3.7 (1.1–14.1)	0.025
Social support		0.401
High	1	
Low	1.2 (0.8–1.8)	
Diagnosis of COVID-19		0.562
Negative	1	
Positive	1.3 (0.5–3.2)	
